# A Web-Based Cancer Self-Management Program (I-Can Manage) Targeting Treatment Toxicities and Health Behaviors: Human-Centered Co-design Approach and Cognitive Think-Aloud Usability Testing

**DOI:** 10.2196/44914

**Published:** 2023-07-21

**Authors:** Doris Howell, Denise Bryant Lukosius, Jonathan Avery, Athina Santaguida, Melanie Powis, Tina Papadakos, Vincenzo Addario, Mike Lovas, Vishal Kukreti, Kristen Haase, Samantha J Mayo, Janet Papadakos, Saeed Moradian, Monika K Krzyzanowska

**Affiliations:** 1 Department of Supportive Care Princess Margaret Cancer Research Institute Toronto, ON Canada; 2 School of Nursing McMaster University Hamilton, ON Canada; 3 Department of Medical Oncology Juravinski Cancer Centre Hamilton, ON Canada; 4 School of Nursing University of British Columbia Vancouver, ON Canada; 5 Ontario College of Art and Design University of Toronto Toronto, ON Canada; 6 Faculty of Nursing University of Toronto Toronto, ON Canada; 7 Faculty of Nursing York University Toronto, ON Canada

**Keywords:** web-based program, self-management, cancer treatment, digital technology, co-design, usability

## Abstract

**Background:**

Patients with cancer require adequate preparation in self-management of treatment toxicities to reduce morbidity that can be achieved through well-designed digital technologies that are developed in co-design with patients and end users.

**Objective:**

We undertook a user-centered co-design process in partnership with patients and other knowledge end users to develop and iteratively test an evidence-based and theoretically informed web-based cancer self-management program (I-Can Manage). The specific study aims addressed in 2 phases were to (1) identify from the perspective of patients with cancer and clinicians the desired content, features, and functionalities for an online self-management education and support (SMES) program to enable patient self-management of treatment toxicities (phase 1); (2) develop the SMES prototype based on human-centered, health literate design principles and co-design processes; and (3) evaluate usability of the I-Can Manage prototype through user-centered testing (phase 2).

**Methods:**

We developed the I-Can Manage program using multiperspective data sources and based on humanistic and co-design principles with end users engaged through 5 phases of development. We recruited adult patients with lung, colorectal, and lymphoma cancer receiving systemic treatments from ambulatory clinics in 2 regional cancer programs for the qualitative inquiry phase. The design of the program was informed by data from qualitative interviews and focus groups, persona and journey mapping, theoretical underpinnings of social cognitive learning theory, and formalized usability testing using a cognitive think-aloud process and user satisfaction survey. A co-design team comprising key stakeholders (human design experts, patients/caregiver, clinicians, knowledge end users, and e-learning and digital design experts) was involved in the developmental process. We used a cognitive think-aloud process to test usability and participants completed the Post-Study System Usability Questionnaire (PSSUQ).

**Results:**

In the initial qualitative inquiry phase, 16 patients participated in interviews and 19 clinicians participated in interviews or focus groups and 12 key stakeholders participated in a persona journey mapping workshop to inform development of the program prototype. The I-Can Manage program integrates evidence-based information and strategies for the self-management of treatment toxicities and health-promoting behaviors in 6 e-learning modules (lay termed “chapters”), starting with an orientation to self-management. Behavioral exercises, patient written and video stories, downloadable learning resources, and online completion of goals and action plans were integrated across chapters. Patient participants (n=5) with different cancers, gender, and age worked through the program in the human factors laboratory using a cognitive think-aloud process and all key stakeholders reviewed each chapter of the program and approved revisions. Results of the PSSUQ (mean total score: 3.75) completed following the cognitive think-aloud process (n=5) suggest patient satisfaction with the usability of I-Can Manage.

**Conclusions:**

The I-Can Manage program has the potential for activating patients in self-management of cancer and treatment toxicities but requires testing in a larger randomized controlled trial.

## Introduction

### Background

The burden of cancer and its treatment is a major cause of morbidity and growing health care costs worldwide [1,2]. Systemic therapies remain highly effective treatments for cancer [3] but are associated with a myriad of treatment-related toxicities, including fatigue, myalgia, gastrointestinal disturbances (nausea, vomiting, diarrhea), that can range from mild and temporary to severe, chronic, and debilitating [4-6]. Treatment toxicities (also called treatment side effects) are highly distressing [7], can lead to poor treatment adherence [8], and high rates of costly emergency department visits [9-11]. Ultimately, it is patients and their caregivers that shoulder responsibility for self-management (SM) of treatment toxicities and the effects of cancer at home between clinic visits with minimal support from health care providers. Access to high-quality education tools and resources that enable patients to effectively manage complex treatment-related toxicities in routine care are lacking [12,13], leaving patients vulnerable to potentially life-threatening severe adverse events, poorer functioning in daily life, long-term disability, and possibly worse survival [14,15].

Similar to the posttreatment survivorship phase [16], the acute treatment phase of cancer should be considered a “teachable moment” in which self-management education and support (SMES) are leveraged to optimize patients’ well-being and strengthen their use of core SM skills (ie, goal setting/action plans, problem solving, decision-making, communication with providers, self-monitoring) [17] and behaviors specific to treatment side effect management. SM is defined as involving the day-to-day tasks, problem-specific strategies, and behaviors individuals must undertake for self-monitoring and management of their disease and symptoms [18]. People living with cancer often feel anxious, overwhelmed, and confused by the sheer volume of information and medical jargon they must digest [19] and need educational materials, including verbal instructions augmented by written documentation, and multimedia learning tools to support their learning and retention [20,21]. In this context, SMES that enables patients to gain self-efficacy in the use of core SM skills (ie, goal setting/action plans, problem solving, decision-making, communication with providers, self-monitoring) [22,23] and behaviors specific to toxicity management and to optimize health [24] are essential early in the diagnosis and treatment phase of cancer and across the cancer trajectory.

Digital technologies are fast emerging for the delivery of SMES for chronic illness outside the walls of hospitals and clinics [25] and are a necessity in the context of constrained health care resources [26]. Digital delivery of SMES is also timely in the context of the COVID-19 pandemic, which has helped put in place the infrastructure necessary to support virtual care [27]. Systematic reviews of digital self-management interventions (DSMIs) in cancer populations show benefits for reducing symptom severity and improving quality of life [28,29]. However, heterogeneity in SMES interventions [30] and what should be translated into DSMIs support components and functionalities has led to some uncertainty about effectiveness [31]. Moreover, many DSMIs are developed without a guiding theoretical framework [32,33], focus on passive dissemination of information [34], and seldom include functionalities that promote patient activation or application of SM behaviors and uptake of health behaviors. Many have not been developed using a co-design approach or best practices in usability testing and seldom target the active treatment phase of cancer [35]. DSMIs seldom focus on active involvement of patients in the early SM of cancer and treatment toxicities [36] or develop programs that address eHealth literacy, which plays a significant role in the uptake of health interventions [37].

### Objectives

Our work addresses these gaps in digital SM programs through the development of the I-Can Manage program, an evidence-based and theoretically informed online SMES program that targets the acute diagnostic and systemic treatment phase of cancer. The overall aim of this study was to ensure usability, uptake, and potential effectiveness of the I-Can Manage program through engagement of patient partners and knowledge end users in its co-design. The specific study aims addressed in 2 phases were to (1) identify from the perspective of patients with cancer and clinicians the desired content, features, and functionalities for an online SMES program to enable patient SM of treatment toxicities (phase 1); (2) develop the SMES prototype based on human-centered [38], health literate design principles [37] and co-design processes [39]; and (3) evaluate initial usability of the I-Can Manage prototype through user-centered testing (phase 2). In this paper, we describe the development of the I-Can Manage prototype, co-design approach, and results of usability testing.

## Methods

### Study Design

**Figure 1:**
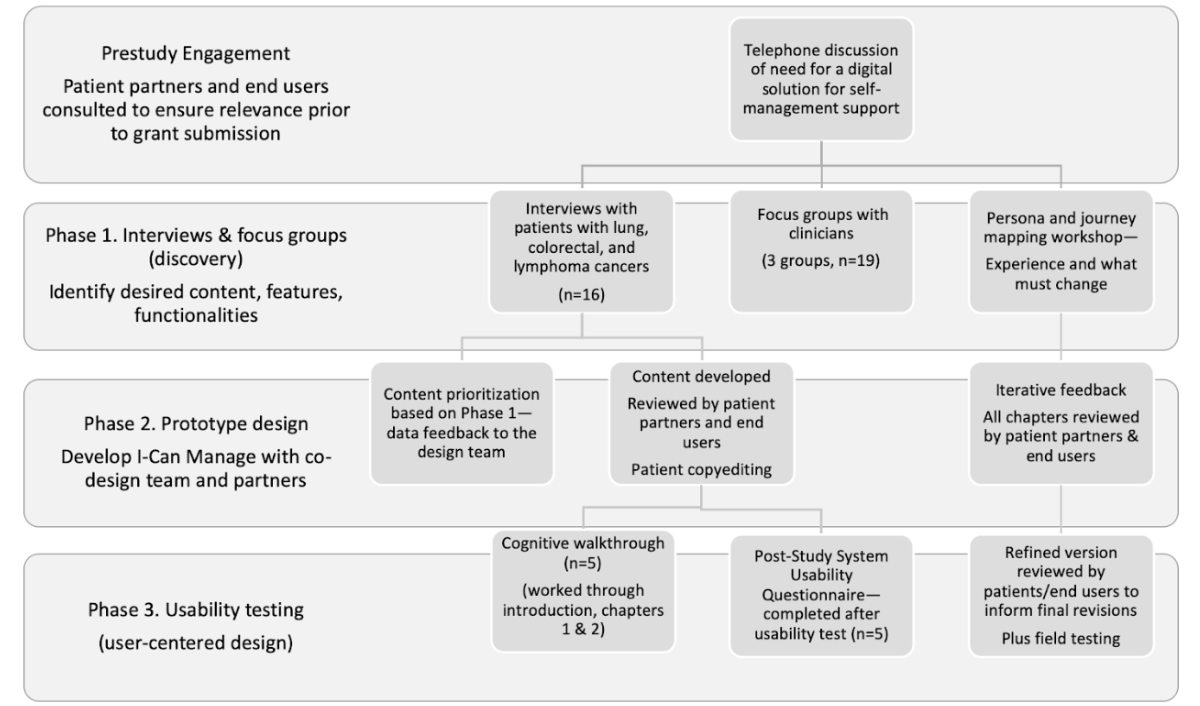
Multiperspective data sources.

The overall study design was descriptive, sequential mixed methods (qualitative interviews and usability survey) [40]. Study participants (patients and clinicians) were recruited from ambulatory cancer clinics at a large comprehensive cancer, Princess Margaret Cancer Centre (Toronto, ON), and a regional cancer program (Juravinski Cancer Centre, Hamilton, ON). The methods and qualitative data insights (results) are presented together reflecting the iterative nature of the co-design process and the multiple perspectives that informed development of the I-Can Manage program. The overall co-design approach and the multisources of data that informed the prototype development are shown in Figure 1. The specific methods and results are further elaborated for each phase of the development and design below.

### Ethical Considerations

The study was approved by the Research Ethics Board of the University Health Network (Toronto, ON; 17-5533.7) and the Juravinski Cancer Center (Hamilton, ON; 3624). Written informed consent was provided by all participants (patients, clinicians) recruited for the qualitative interviews, focus groups, and usability testing phase of the prototype development. Demographic information was collected from participants including age, type of cancer, gender, date since last treatment, type of treatment received, and comfort with use of digital technology. All data were deidentified including the data obtained in a persona mapping workshop that included members of the design team. Patient participants in the design team meetings and usability testing phase of the study received reimbursement for travel expenses and parking costs. We adhered to local, national, regional, and international law and regulations regarding protection of personal information, privacy, and human rights as required for digital technology. Ethics approval number for this study is 17-5533.7.

### Overview of the Development Process

**Figure 2:**
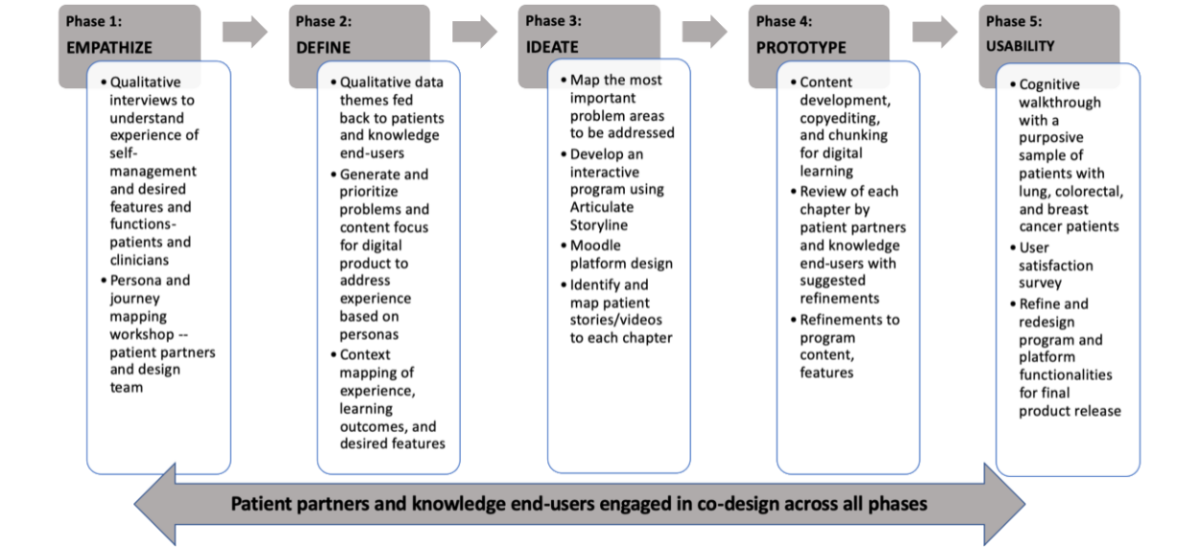
Phases of human centred and co-design approach.

As shown in Figure 2, we followed a 5-phase human-centered and co-design thinking process [41,42] to develop the I-Can Manage program with engagement of patients as end user partners and other knowledge end users (eg, clinicians, administrators, cancer support service leaders) throughout all stages of the research process from inception of the research question to prototype completion. Persons with lived experience of cancer or their caregivers interested in participating in the research were recruited from a provincial cancer agency through an email blast sent from the program administrator to their list of volunteers as patient partners. The email blast described the proposed research and invited them to engage as partners in the co-design of the digital SM learning platform, I-Can Manage. Interested patients or caregivers were then contacted by telephone to further discuss the proposed research and confirm their interest in participating and the need for the proposed program.

Our design team comprised 6 patients (3 females with breast cancer [1 Aboriginal], 2 males with lymphoma, and 1 male with lung cancer), 1 caregiver, 3 digital designers, 2 clinicians (nurses), 1 PhD student experienced in adolescents and young adults populations, and knowledge end users (medical oncologist, national cancer services director, cancer information specialist, eHealth literacy expert, and experts in patient education) with diverse cancer experiences who were engaged to provide input throughout the program development and design phases.

### Phases of Development and Design

#### Phase 1: Empathize

To understand user needs in the empathize phase, a qualitative inquiry was conducted based on a qualitative descriptive methodology [43]. The goal of qualitative description is to provide a rich description of an experience in an easily understandable language and focus on who, what, how, and where questions regarding a phenomenon of interest (ie, SM of treatment side effects). It is particularly suited for health service research [44]. The goal of the qualitative inquiry was to gain insights into the experience of patients with lymphoma, lung, and colorectal cancer (n=16) and clinicians (n=19) on SM, views of desired content, features/functionalities, and optimal timing for the SMES program during systemic treatment. The full methods and results of the qualitative inquiry were previously published [45].

#### Patient Recruitment

Briefly, adult patients (aged ≥18 years) with lymphoma, lung, and colorectal cancer were recruited from ambulatory cancer clinics in a comprehensive cancer center and regional cancer program if they met the study eligibility criteria (not more than 3 months from the completion of systemic cancer treatment or currently receiving systemic cancer treatment, English speaking, Eastern Cooperative Oncology Group status of 0-2 such that self-care was possible, familiar with the internet or use of a phone). Potentially eligible patients were identified by members of the circle of care. Clinicians were invited to participate using email correspondence sent from their program manager and included oncologists, nurses, social workers, allied health, and psychologists. Individual qualitative interviews were conducted with patients and focus groups were conducted with clinicians.

### Qualitative Data Insights

#### Overview

**Figure 3:**
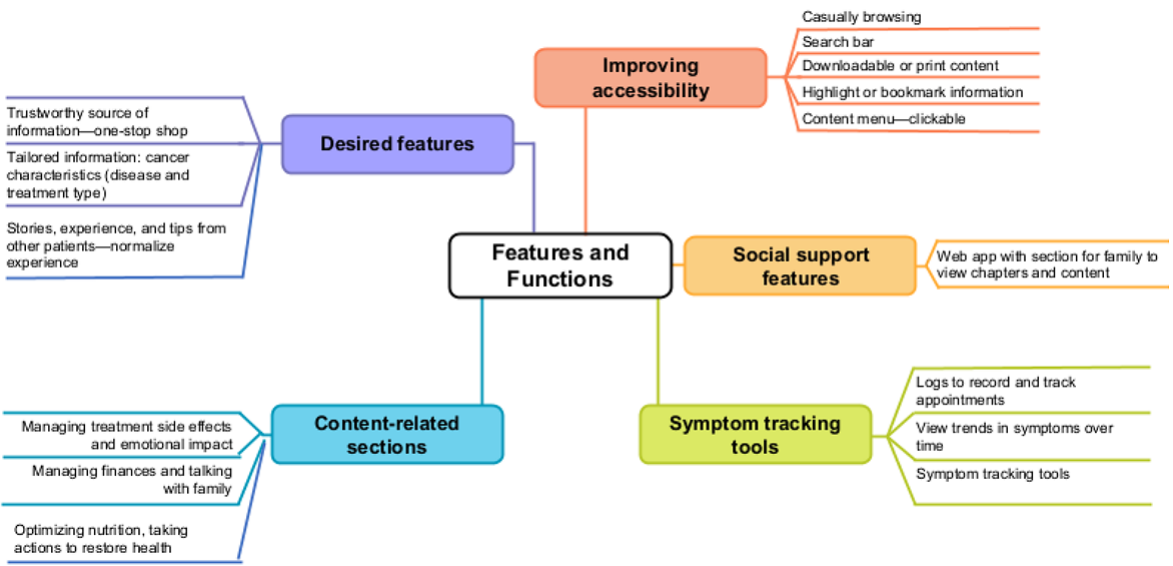
Desired features and functions.

Results that are highlighted herein are to show how these data informed the design of I-Can Manage and to ensure we took into consideration the patients’ experience of cancer as essential for a humanistic design approach. Briefly, analysis of the data revealed managing cancer and treatment as “hard work.” Patients wanted information tailored to their personal context, to learn from other patients with cancer in the hopes of “normalizing” their experience, and support for managing emotional consequences, which they reported as “neglected” in the active treatment phase of cancer. For instance, as part of normalizing the experience, in the treatment toxicity module we described the experience of symptoms from the perspective of patients as a way to normalize the experience (ie, myalgia feels like aching in the bone and joints). The desired features and functionalities derived from qualitative interviews are shown here to establish the context for how I-Can Manage was designed (Figure 3).

Following the qualitative inquiry, patients, caregivers, clinicians, knowledge end users, and digital design experts (n=12) were invited to a persona mapping workshop. Persona mapping is the creation of fictional, but realistic profiles of the users of the program and their journey [46]. In the persona mapping workshop, participants were engaged in persona and cancer experience journey mapping to develop a deeper understanding of their real-world experience of being diagnosed with cancer, managing treatment toxicities, and participating in health recovery; their perspectives of what they needed to know and their hopes for a digital solution to improve their experience; and the challenges they experienced.

Most participants in the persona mapping workshop were patients (n=6) and caregiver (n=1), clinicians (n=2), designers (n=2), and knowledge end users (n=1) who were able to attend (reimbursed for travel) from our design group described earlier in the paper. Groups of patient partners, clinicians, end users were mixed in small working groups to ensure all perspectives were voiced and heard and a facilitator was assigned to each group. Participants described the defining moments that stood out across phases of their cancer journey and care. These defining moments included experiences of not knowing how to talk to family and friends about the diagnosis or not knowing whether their emotional reactions or treatment side effects were normal, and the devastating effects of dealing with the life-altering nature of cancer (Multimedia Appendix 1). One participant remembering the day of diagnosis stated, “today my life has changed.”

Participants were asked to describe what they needed to learn, know, and do. Participants also expressed the need for a single source of trustworthy information to manage treatment side effects, to learn from experiences of other patients, and to know what actions they could take to help themselves to persevere, endure cancer and treatment, and recover health. Persona data were then used to inform the content of the I-Can Manage program. This was operationalized as co-design by sharing key topics and desired content based on qualitative data that were provided back to participants and our design team before we further developed the program to ensure the content “held true” for their experience and to identify “SM support needs” to be met by our digital solution. For example, in this workshop, participants were asked about what they needed to know and understand about how to tell their family regarding their diagnosis as a defining moment and what they hoped could change with the online program. Thus, for example, in module (chapter) 1 we provided specific information about sharing the diagnosis and considerations for “who” and “how to tell” (eg, a work colleague vs a young child or older child) and this was linked to patient’s stories and other resources about how to talk to family members including children about a cancer diagnosis.

#### Phase 2: Define

In addition to data from the persona and journey mapping, themes from qualitative data were reported back to the design team. Consequently, core problems in managing cancer treatment toxicities and side effects were identified and key content and features for the “I-Can Manage” program to address these problems were prioritized for inclusion by the design team. Additionally, an experienced oncology nurse (DH) developed content based on her experience and knowledge of working with cancer populations and evidence-based guidelines [47,48], including patient versions of symptom SM guidance documents [49]. A patient partner experienced in digital design was engaged to further chunk content for digital delivery and copyediting to ensure use of plain language and a coherent flow of information. The design team was involved in iterative development and design cycles that included reviewing and providing feedback for each program chapter to inform refinements and iterative development.

#### Phase 3: Ideate

The I-Can Manage program was specifically designed to target the active treatment phase of cancer. Using what we learned from these multiperspective data sources including the qualitative inquiry and the journey mapping workshop, we developed a context-mapping approach [41] to develop a framework that would guide development of the prototype, expected learning outcomes, features, and functions (Table 1).

**Table 1:** The I-Can Manage components at a glance to address patient experience from multiperspective data sources, including behavioral exercises to build self-management skills/efficacy.

Focus	Module 1: Regaining your balance	Module 2: Managing treatment side effects	Module 3: Coping with stress and emotions	Module 4: Balancing fatigue and activities	Module 5: Optimizing health and quality of life
Patient experience	Shock and crisis Fears of incapacitation/death/telling family Information overload Psychosocial impact forgotten in the acute phase	Anxiety/fears about treatment and side effects. What do they feel like Concerns about the effectiveness of treatment Anticipating/managing side effects. What works for recovery	Stress/roller coaster of emotions Feelings of uncertainty/sense of vulnerability Need new ways of coping Change in roles and family/friend relationships	Overwhelmed with fatigue Vicious cycles of fatigue, rest, deconditioning, insomnia, PA^a^ Adjust PA to acute treatment effects	Interrupted functioning in daily life and work Illness intrusiveness, self-esteem, body image, sexual health, relationships Restoring quality of life
Learning outcomes	Knowledge of emotional reactions to diagnosis Able to identify desired role as partner in health care and personal strengths Able to apply mindful breathing to reduce anxiety early in diagnosis Confident in communication with family/friends/providers	Knowledge about what to expect regarding chemotherapy side effects/normal pattern of side effects/recovery Able to differentiate between normal and adverse effects to report to providers (health team) Apply symptom self-monitoring for tailoring daily behaviors Confident in the use of self-management behaviors to prevent/reduce side effects	Knowledge of physiological reactions to stress and mind/body connections Able to apply positive coping skills and problem-solving to manage emotions and uncertainty Able to differentiate between normal emotions and depression/anxiety to report to providers Confident in the use of stress-reducing behaviors	Knowledge of energy-bank model of fatigue and body capacity Able to differentiate between usual and cancer fatigue Able to apply energy conservation and adaptive pacing in daily life Confident in the use of behaviors and PA to manage fatigue	Knowledge of healthy lifestyle behaviors and influence on cancer recovery Taking action on smoking cessation and health behaviors Able to apply healthy eating to manage specific problems (eg, weight gain or loss) Confident in the use of behaviors to optimize quality of life
Select program content	Regaining your balance Strategies to manage initial anxiety and fear Desired role in and making decisions aligned with health values Forming a partnership with health team Being effective in self-management Mobilizing personal strengths/support systems Talking with others about your diagnosis	Overview of chemotherapy and other types of cancer treatment Chemotherapy side effects (pattern, type, normalize how they feel, self-management strategies/specific behaviors to reduce effects on daily function) Titrating medications to optimize effectiveness Avoiding and recognizing signs and symptoms of infection Adjusting work and life activities What can family and friends do to support you through treatment	Stress and crisis reactions Reframing of beliefs about illness Normalize emotional turbulence and emotional reactions (emphasize positive emotions) Practical tools for coping (ie, relaxation, mindfulness, meditation, self-talk) Mobilizing peer support Breaking vicious cycles of negative emotions and symptoms Application of positive coping skills including problem-solving	Adaptive pacing for energy conservation Graded physical activity (avoid boom and bust) to tolerance Developing a physical activity plan during treatment Breaking vicious cycles of fatigue, rest, and insomnia Scaling fatigue for self-management and behavior adjustment Application of sleep hygiene to address insomnia	Recognizing health values Healthful nutrition during treatment Taking action on healthy lifestyle behaviors Restoring quality of life, putting wellness in the foreground Restoring meaning and purpose in life/leisure activities Adjusting to change in work, vocational, and other life roles Dealing with cancer worry/fear of recurrence Sexuality and intimacy
Behavioral exercises to build core self-management skills and self-efficacy	Recognizing your personal strengths and resources Vicarious learning (deep breathing, active relaxation, positive self-talk) What is your decision-making style tool? How to make decisions Partner in health scale Decision balance tool (weighing the pros and cons of treatment options) Goal and action plan	Self-monitoring of symptoms and side effects; tracking severity with sliding scale and graph over time Self-assessment of confidence in managing treatment side effects Daily decisions (eg, adherence to medications) Tailoring of behaviors to manage effects Specific strategies for managing common treatment side effects Goal and action plan	Recognize and manage your cancer stressors worksheet Dealing with anxiety and panic (5-4-3-2-1 exercise) Recognizing and breaking vicious cycles between your thoughts, emotions, and behaviors Self-assessment of coping skills and which skills to strengthen Problem-solving practice worksheet Building on your coping skills Goal and action plan	Problem-solving barriers to activity Developing your FITT^b^-graded physical activity plan Scaling severity of fatigue using a 0-10-word scale Monitoring fatigue using a daily diary for adjusting physical activity Using a Perceived Exertion Scale Goal and action plan	Identify your 4 quadrants of quality of life A balanced life (the wellness wheel) Build a healthy eating plan Goal and action plans

^a^PA: physical activity.

^b^FITT: frequency, intensity, time, and type.

Additionally, the design of the platform adhered to eHealth literacy principles including intuitive navigation, plain language, and iterative testing with end users. The design of the I-Can Manage program was theoretically underpinned by social cognitive learning theory and the construct of self-efficacy, which relates to an individual’s belief in their own capability [50]. This was achieved by incorporating action-oriented information and behavioral exercises as a core feature of the program to promote application of core SM skills (ie, problem solving, goal setting/taking action, decision-making, symptom self-monitoring using tracking and diaries, resource use, collaborative communication, and partnering with health care providers) [22], problem-specific SM strategies (ie, physical activity for fatigue), and health behaviors to optimize health and wellness (ie, eating healthy, smoking cessation).

Program features incorporated the 4 main sources of self-efficacy information [51] including (1) mastery learning (ie, symptom self-monitoring and learning how to adjust behaviors based on symptom severity, goal setting/action planning); (2) vicarious experiences through the inclusion of videos that modeled a behavior (ie, how to do mindful breathing, physical activity demonstrations, and patients talking about emotions and how they coped); (3) social persuasion through the inclusion of patient stories (video and written) and also to normalize their experience (ie, what does cancer fatigue feel like); and (4) emotional and physiological states through the inclusion of downloadable patient exercises to identify personal strengths and coping skills, reframing negative emotional states, and breaking vicious symptom cycles (ie, avoiding boom and bust in managing cancer fatigue).

#### Phase 4: Prototype

**Figure 4:**
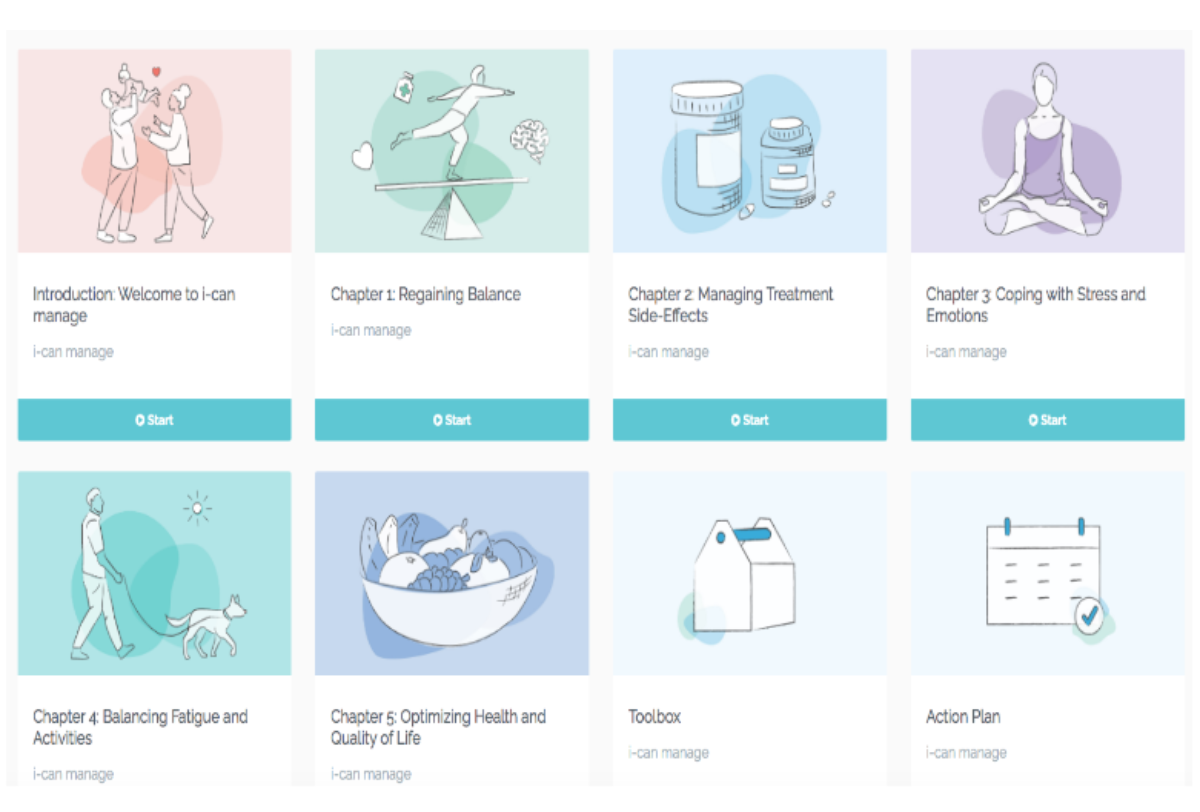
Overview of the I-Can Manage program.

**Figure 5:**
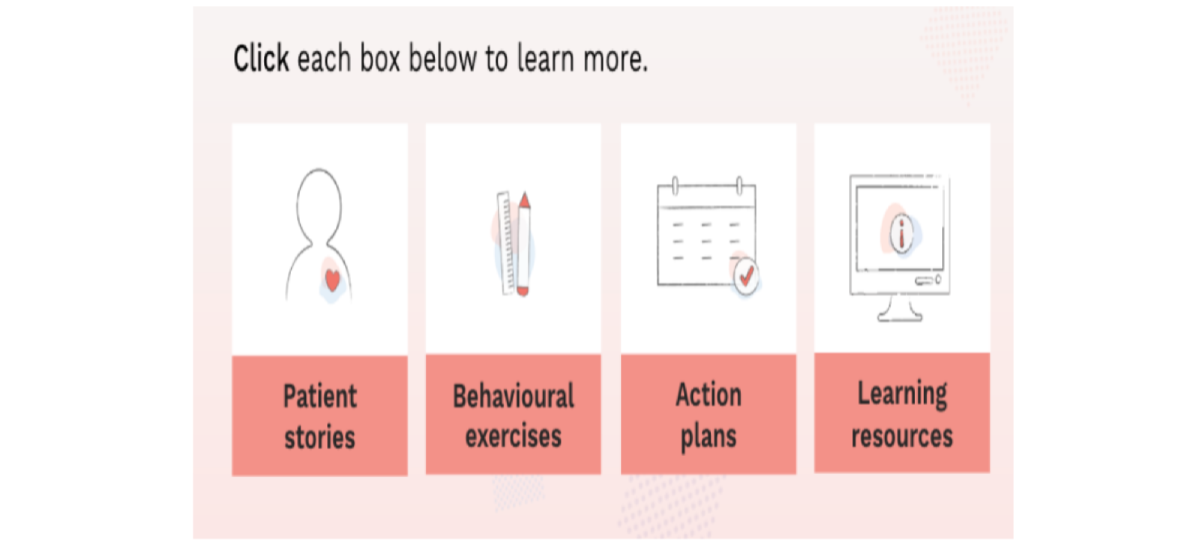
Cross-cutting elements for each chapter to support behavior change.

We used the e-learning authoring software Articulate 360 storyline [52] to produce interactive and video-based content; and the Moodle learning management system (Moodle Community) [53] as the web-based learning platform to host the content, register patients, and track usage patterns. Links to other sites were integrated for deeper learning and to facilitate tailoring of information to the needs of user and to ensure access to trustworthy information sources such as the American Society of Clinical Oncology (ASCO; clinician-approved patient fact sheets) [54]. The final prototype of the I-Can Manage web-based program comprised a welcome and introductory chapter, and 5 learning chapters with 4-5 sections per chapter (Figure 4), and core elements to support uptake of SM strategies and behavior change supported through completion of downloadable worksheets embedded in each chapter (Figure 5). Participants are given suggestions at the start of the program as to what modules they may want to complete first and the order of the modules. However, the program allowed patients to move around the modules as they desired, as we learned in usability testing that patients want to skip modules that were not relevant to them and to complete modules in a particular order relevant to their needs. Additionally, they desired modules to be open and accessible throughout their cancer treatment journey (ie, not locked so they have to complete 1 chapter to get to the next chapter). For instance, some patients described only scanning the treatment toxicities module as this was a recurrent cancer and so wanted to go directly to the chapter on coping as this was more relevant. As described earlier, each chapter was developed to incorporate core elements and functionalities that focused on building knowledge and use of behaviors to address the multifaceted medical, emotional, and lifestyle tasks of cancer SM [55]. Best practices were used for providing interactive content that would facilitate uptake of SM strategies, behaviors, and behavior change [35]. I-Can Manage emphasizes building of self-efficacy and core SM skills including completion of goal setting and action planning and activation of behaviors including symptom self-monitoring skills using symptom severity scales to inform daily tailoring and adjustment of behaviors.

Thus, we incorporated learning to support use of SM core skills and build self-efficacy including (1) evidence-based content about the best practice strategies and behaviors to manage treatment side effects; (2) patient stories in written and video formats interspersed throughout the program to humanize and normalize their experience, and to deepen their learning about how to apply SM strategies and behaviors to manage treatment toxicities and the emotional effects of cancer; (3) behavioral exercises to build core SM skills (ie, goal setting and action planning); and (4) links to other trustworthy sources such as the ASCO and the Canadian Cancer Society or peer support networks for access to additional support. In addition to evidence-based information, tips for managing treatment side effects from peers and their descriptions of how a symptom felt were used to normalize symptoms and enable patients to hear the voice of experienced others who had traveled this journey before them. Select screenshots portraying a selected sample of the components of Chapter 4 “Balancing Fatigue and Activities” are shown in Multimedia Appendices 2 and 3. Additionally, we show a sample of a downloadable work sheet for tracking cancer fatigue (Multimedia Appendix 4) and one of the number of downloadable education information sheets (Multimedia Appendix 5).

#### Phase 5: Usability (User Interface/User Experience Design) Testing

We used convenience sampling to recruit adult patients (aged 18 and over) diagnosed with diverse cancers (breast, lymphoma, and colorectal; n=5) at a large comprehensive cancer center for participation in usability testing in the digital design laboratory. The eligibility criteria were the same as the qualitative inquiry phase of the study and we attempted to purposively sample for maximal variation in age, type of cancer, gender, culture, and race. Nearly 80% of usability problems can be identified with 5 participants, which is considered an adequate sample for usability testing [56]. Ideally, it is recommended that maximal variation in sampling for usability testing be used in digital design to ensure the program addresses usability from the perspective of different users with different cancer diagnoses, ages (younger and older), gender, diverse cultures, and race. Prospective participants were not previously exposed to the program and agreed to take part in this formalized user interface/user experience design testing using a cognitive walkthrough of the program in the laboratory.

A cognitive walkthrough was used to identify user experiences related to how the content was presented and ease of use of functionalities [57], with user experiences and feedback manually recorded in a spreadsheet. Participants worked through the program in our human factors digital laboratory while being observed and made comments aloud as they worked through the program that was recorded verbatim by the observer. Assigned task included talking aloud to reflect the participants thoughts while working through the “Introduction: Welcome to I-Can Manage” (overview of the program and orientation to SM and their role) and the first 2 chapters (Chapter 1: “Regaining Balance” and Chapter 2: “Managing Treatment Side-Effects”). Participants were also instructed to use the Hamburger Menu and other design elements (buttons to click through the program; Moodle Platform) to navigate to other program chapters in order to determine ease of use of the program. These data were used to inform refinements to the program and finalize the prototype (Multimedia Appendix 6). The Moodle learning platform was further optimized based on usability feedback.

## Results

Usability phase participants ranged in ages from 53 to 67 years; most were married, college/university educated, had diverse cancers, and all were comfortable using the internet (Table 2).

Suggestions for refinements to the program focused mainly on improving navigation through the program (eg, more visible hamburger menu), reducing the number of clicks to move forward in the program, and enabling users to go back to chapters. Additionally, suggestions were made for minor changes to the program content such as lessening the amount of content on each page. Participants described the content as relevant and engaging, but thought there was a lot of content, so we further chunked or eliminated content and placed reminders throughout the program for patients to take a break; and added information about how long it would take to complete chapters so that patients could plan accordingly (Multimedia Appendix 2). Besides, our patient partners and knowledge end users were given open access to the program and asked to provide feedback on each chapter and its content. Their feedback was also used to further refine the program prior to release of the prototype. A specific member of our team did field testing of the prototype across computers and iPads (Apple Inc.) to check links and functionality and to ensure fixes prior to final product release.

Usability patient participants (n=5) also completed the Post-Study System Usability Questionnaire (PSSUQ) [58] to assess their experience and perceived usability of the I-Can Manage program on a 7-point Likert scale of Strongly Agree to Strongly Disagree, with a lower score denoting greater performance and satisfaction with usability of the system. The global mean score for the PSSUQ was 3.75 and for system usefulness, information quality, interface quality this was 3.54, 4.1, and 3.5, respectively (Table 3).

**Table 2:** Participant characteristics for usability testing (N=5).

Characteristic		Frequency
Age (years), mean		50.4
Sex (female), n (%)		3 (60)
Married, n (%)		4 (80)
**Cancer type, n (%)**		
	Breast	2 (40)
	Lung	1 (20)
	Colorectal	1 (20)
	Hematological	1 (20)
**Education, n (%)**		
	High school	1 (20)
	College/university	4 (80)
**Income (CAD^a^), n (%)**		
	<90,000	2 (40)
	>90,001	2 (40)
	Did not want to answer	1 (20)
**Ethnic** **i** **ty, n (%)**		
	Canadian	3 (60)
	Asian	1 (20)
	Jewish	1 (20)
Very comfortable in using the internet, n (%)		5 (100)

^a^CAD $1 =US $0.75.

**Table 3:** Mean scores for the Post-Study System Usability Questionnaire.^a^

Item	Median score	Mean score
Overall score	3.20	3.75
System usefulness	2.83	3.54
Information quality	4.00	4.16
Interface quality	2.75	3.55

^a^Lower scores indicate better performance and satisfaction.

## Discussion

### Summary of Key Results

We developed the content, features, and functions of a web-based SM program, I-Can Manage, iteratively in co-design with patient partners and knowledge end users (eg, cancer peer support program leaders, clinicians) using multiperspective data sources and a 5-phase human-centered development process that included formalized usability testing. The I-Can Manage program was positively viewed by our end users. Satisfaction with the system was high, and it was viewed as an easily navigable SMES program that could be integrated early in the acute diagnosis and acute treatment phase of cancer. Our work addresses a gap in knowledge about the application of an iterative co-design and demonstrates a user-centered digital design process that could be used by other researchers in the development of similar programs.

### Comparison With Prior Work

Digital technology to deliver SMES is increasingly recognized as important for reaching people living with cancer on a wider scale in their own homes and communities and is complementary to guidance by health care professionals and can enhance health system capacity [59,60]. Little research has focused on the potential effectiveness of digitally delivered SMES to enable activation of patients in the SM of treatment-related toxicities, and the psychosocial and lifestyle changes that accompany a cancer diagnosis. A recent review identified 19 studies evaluating DSMIs in cancer populations, with 11 studies focused on the active phase of cancer treatment (population range 34-752 patients) [31]. Most digital programs identified were focused on dissemination of information, patient education, self-care advice, or collection of patient-reported outcomes, symptom data for the purpose of communicating or alerting health care providers versus features and functions to support the application of SM strategies and behaviors. Moreover, there is enormous diversity in intervention content in digital SMES programs and the emphasis placed on uptake of behaviors. Further, many were not developed in co-design with end users or using best practices in usability testing. Thus, not surprisingly, the findings for effectiveness of DSMIs have been mixed, with some studies showing positive effects on quality of life and anxiety and depression, whereas other studies showed no effect. Other systematic reviews showed positive benefits of DSMIs in improving adherence to oral treatment regimens [61], symptom distress [62], and healthy lifestyle behaviors [63]. However, considerable heterogeneity in intervention components tested and outcomes measured are noted for cancer SM interventions in general [32] and for digital programs [59]. Few of these programs include features and functionalities that enable the activation of SM behaviors, core SM skills, and building self-efficacy as key mechanisms for achieving a change in behaviors and improvement in health outcomes [60]. Most focus on dissemination of information or education that may improve knowledge but is inadequate to promote uptake of health behaviors particularly for patients with complex and dynamic illnesses such as cancer. By contrast, I-Can Manage specifically focuses on the behavioral aspects of SM reinforced through the way that information is provided (ie, action oriented) and completion of behavioral exercises by users (eg, steps to follow to develop a graded activity plan, healthy meal plates, breaking vicious cycles of fatigue and negative emotions, coping strategies, goal setting and action planning for each module, and building of self-efficacy).

### Broader Implications

Patient engagement in SM is a desired standard of quality cancer care [64] that has not yet been integrated in routine practice [65] and patients describe poor access to SM in ambulatory care [66]. The I-Can Manage program has universal applicability for systemic (chemotherapy and immunotherapy) or oral cancer treatments as it is agnostic to cancer type. It is intended to capitalize on the diagnosis and treatment phase of cancer as a “teachable moment” to support patients in managing the multiple tasks of cancer and treatment early in the continuum. SM is particularly challenging during the acute phase of cancer because people are learning a new medical language and how to manage toxicities for often complex treatment regimens alongside dealing with the emotional sequalae of cancer, and seldom realize they can take actions to optimize health.

We envision future chapters and functionalities that support tailoring to cancer type, treatment modalities, phases of cancer care (ie, posttreatment survivorship), and differing needs of younger and older patient populations, as well as cultural, ethnic, and race diversity. For example, social media and peer support are considered essential to young adults with cancer [67], and future iterations will need to optimize Moodle functionalities or a native app-based format to offer these components. The I-Can Manage program provided direct links to other reputable organizations whose mandate was peer support. It is also recognized that older individuals may require tailored SM support programs that address multimorbidity [68] and changes in cognitive capacity that occur with aging and can impact on learning [69].

Chronic diseases such as cancer place a significant burden on health care systems globally and are a major source of health care expenditure. Disease SM programs are advocated as a solution to this problem; however, little progress has been made in the redesign of health care systems to ensure integration of these programs in routine cancer care [12]. Digital technology to support patient activation in disease and health SM leveraging programs such as the I-Can Manage program may be more widely scalable than trying to redesign complex care systems, which have largely failed to date [70].

Digital SMES should be considered an essential component of routine clinical care that is financed and integrated in a comprehensive program of SM support [71,72], particularly in the context of episodic ambulatory cancer care that is characterized by high-volume patient loads and short rapid visits without scheduled follow-up for SM training or support. Thus, implementation of SMS in cancer care settings has been challenging and progress lags compared with other chronic diseases. Future research should also identify the essential components of digital SM support solutions that translate into behavior change and clinicians will need to gain comfort in prescribing digital therapeutics as part of their treatment approach; besides, implementation research will be crucial to promote uptake in practice.

Future research should focus on formal testing of the I-Can Manage program on SM behaviors and health outcomes and adaptations for tailoring to different treatment modalities, cancer types, socioeconomic and cultural diversity, and older and younger age groups. We expect that the I-Can Manage program would result in improved self-efficacy, uptake of SM strategies, and better quality of life and wellness that requires testing in a clinical trial.

### Limitations

There are some limitations in our work to consider such as the inclusion of patients and clinicians from only 2 cancer centers in the qualitative inquiry. Although our patient partners were from rural/remote and urban regions, most had high levels of education and income that could have introduced some selection bias in the study. Additionally, convenience sampling was used for recruiting patients for usability testing, the sample size was small, and only patients were included and not caregivers. This could have introduced some selection bias given that those interested in digital solutions or more computer literate may have been more willing to participate. Our usability testing focused on tasks for completion that only included the Introduction, and Chapters 1 and 2 and not all chapters given the time that would be required to complete them in a single session.

### Conclusions

Suboptimal management of cancer treatment toxicities can lead to serious complications and negative effects on quality of life and worse survival. Patients/families require education and SM support to apply the SM strategies and behaviors necessary to effectively manage the medical, emotional, and lifestyle changes that are necessary to adapt to cancer and treatment. Digital SMES is a promising solution that requires future testing for its effectiveness on improving health outcomes.

## Appendix

Multimedia Appendix 1 Summarized themes of experience, defining moments, and needs from persona and cancer journey mapping.

Multimedia Appendix 2 Select screenshots for Chapter 4: "Balancing Fatigue and Activity.".

Multimedia Appendix 3 Additional screenshots for Chapter 4: "Balancing Fatigue and Activity.".

Multimedia Appendix 4 Downloadable fatigue diary for symptom monitoring.

Multimedia Appendix 5 Downloadable information sheet—sleep hygiene behavior tips.

Multimedia Appendix 6 Summary of user feedback.

## Abbreviations

ASCO: American Society of Clinical Oncology
DSMI: digital self-management intervention
PSSUQ: Post-Study System Usability Questionnaire
SM: self-management
SMES: self-management education and support

## Data Availability

Data can be made available upon request to the corresponding author.

## References

[1] Sung H, Ferlay J, Siegel RL, Laversanne M, Soerjomataram I, Jemal A, Bray F (2021). Global Cancer Statistics 2020: GLOBOCAN Estimates of Incidence and Mortality Worldwide for 36 Cancers in 185 Countries. CA Cancer J Clin.

[2] Carlotto A, Hogsett VL, Maiorini EM, Razulis JG, Sonis ST (2013). The economic burden of toxicities associated with cancer treatment: review of the literature and analysis of nausea and vomiting, diarrhoea, oral mucositis and fatigue. Pharmacoeconomics.

[3] Iwamoto T (2013). Clinical application of drug delivery systems in cancer chemotherapy: review of the efficacy and side effects of approved drugs. Biol Pharm Bull.

[4] Kerckhove N, Collin A, Condé Sakahlé, Chaleteix S, Pezet D, Balayssac D (2017). Long-Term Effects, Pathophysiological Mechanisms, and Risk Factors of Chemotherapy-Induced Peripheral Neuropathies: A Comprehensive Literature Review. Front Pharmacol.

[5] Aprile G, Rihawi K, De Carlo Elisa, Sonis S (2015). Treatment-related gastrointestinal toxicities and advanced colorectal or pancreatic cancer: A critical update. World J Gastroenterol.

[6] Reilly CM, Bruner DW, Mitchell SA, Minasian LM, Basch E, Dueck AC, Cella D, Reeve BB (2013). A literature synthesis of symptom prevalence and severity in persons receiving active cancer treatment. Support Care Cancer.

[7] Lehto U, Tenhola H, Taari K, Aromaa Arpo (2017). Patients' perceptions of the negative effects following different prostate cancer treatments and the impact on psychological well-being: a nationwide survey. Br J Cancer.

[8] DiMatteo M, Haskard K, Williams S (2007). Health beliefs, disease severity, and patient adherence: a meta-analysis. Med Care.

[9] Mayer DK, Travers D, Wyss A, Leak A, Waller A (2011). Why do patients with cancer visit emergency departments? Results of a 2008 population study in North Carolina. J Clin Oncol.

[10] Lash RS, Bell JF, Reed SC, Poghosyan H, Rodgers J, Kim KK, Bold RJ, Joseph JG (2017). A Systematic Review of Emergency Department Use Among Cancer Patients. Cancer Nurs.

[11] Roy M, Halbert B, Devlin S, Chiu D, Graue R, Zerillo JA (2021). From metrics to practice: identifying preventable emergency department visits for patients with cancer. Support Care Cancer.

[12] Howell D, Mayer D, Fielding R, Eicher M, Verdonck-de Leeuw Irma M, Johansen C, Soto-Perez-de-Celis E, Foster Claire, Chan Raymond, Alfano Catherine M, Hudson Shawna V, Jefford Michael, Lam Wendy W T, Loerzel Victoria, Pravettoni Gabriella, Rammant Elke, Schapira Lidia, Stein Kevin D, Koczwara Bogda, Global Partners for Self-Management in Cancer (2021). Management of Cancer and Health After the Clinic Visit: A Call to Action for Self-Management in Cancer Care. J Natl Cancer Inst.

[13] Hammer M, Ercolano E, Wright F, Dickson V, Chyun D, Melkus G (2015). Self-management for adult patients with cancer: an integrative review. Cancer Nurse.

[14] Jairam V, Lee V, Park HS, Thomas CR, Melnick ER, Gross CP, Presley CJ, Adelson KB, Yu JB (2019). Treatment-Related Complications of Systemic Therapy and Radiotherapy. JAMA Oncol.

[15] Panagioti M, Richardson G, Small N, Murray E, Rogers A, Kennedy A, Newman S, Bower P (2014). Self-management support interventions to reduce health care utilisation without compromising outcomes: a systematic review and meta-analysis. BMC Health Serv Res.

[16] Frazelle ML, Friend PJ (2016). Optimizing the Teachable Moment for Health Promotion for Cancer Survivors and Their Families. JADPRO.

[17] Center for the Advancement of Health (2022). Essential Elements of Self-Management Interventions.

[18] Clark NM, Becker MH, Janz NK, Lorig K, Rakowski W, Anderson L (2016). Self-Management of Chronic Disease by Older Adults. J Aging Health.

[19] Sørensen Kristine, Makaroff LE, Myers L, Robinson P, Henning GJ, Gunther CE, Roediger AE (2020). The call for a strategic framework to improve cancer literacy in Europe. Arch Public Health.

[20] Valenti RB (2014). Chemotherapy education for patients with cancer: a literature review. Clin J Oncol Nurs.

[21] Apor E, Connell NT, Faricy-Anderson K, Barth P, Youssef R, Fenton M, Sikov WM, Thomas A, Rosati K, Schumacher A, Lombardo A, Korber S, Khurshid H, Safran H, Mega A (2018). Prechemotherapy Education: Reducing Patient Anxiety Through Nurse-Led Teaching Sessions. Clin J Oncol Nurs.

[22] Lorig KR, Holman HR (2003). Self-management education: history, definition, outcomes, and mechanisms. Ann Behav Med.

[23] Bodenheimer T, Lorig Kate, Holman Halsted, Grumbach Kevin (2002). Patient self-management of chronic disease in primary care. JAMA.

[24] Foster C, Fenlon D (2011). Recovery and self-management support following primary cancer treatment. Br J Cancer.

[25] Marthick Michael, McGregor Deborah, Alison Jennifer, Cheema Birinder, Dhillon Haryana, Shaw Tim (2021). Supportive Care Interventions for People With Cancer Assisted by Digital Technology: Systematic Review. J Med Internet Res.

[26] Emanual DME, Steinmetz A, Schmidt H (2019). Rationing and resource allocation in health care: essential readings. Theor Med Bioeth.

[27] Gabryelczyk R (2020). Has COVID-19 Accelerated Digital Transformation? Initial Lessons Learned for Public Administrations. Information Systems Management.

[28] Escriva Boulley G, Leroy T, Bernetière Camille, Paquienseguy F, Desfriches-Doria O, Préau Marie (2018). Digital health interventions to help living with cancer: A systematic review of participants' engagement and psychosocial effects. Psychooncology.

[29] Kim AE, Park Hyeoun-Ae (2015). Web-based Self-management Support Interventions for Cancer Survivors: A Systematic Review and Meta-analyses. Stud Health Technol Inform.

[30] Howell D, Harth T, Brown J, Bennett C, Boyko S (2017). Self-management education interventions for patients with cancer: a systematic review. Support Care Cancer.

[31] Aapro M, Bossi P, Dasari A, Fallowfield L, Gascón P, Geller M, Jordan K, Kim J, Martin K, Porzig S (2020). Digital health for optimal supportive care in oncology: benefits, limits, and future perspectives. Support Care Cancer.

[32] Cuthbert CA, Farragher JF, Hemmelgarn BR, Ding Q, McKinnon GP, Cheung WY (2019). Self-management interventions for cancer survivors: A systematic review and evaluation of intervention content and theories. Psychooncology.

[33] Corbett T, Singh K, Payne L, Bradbury K, Foster C, Watson E, Richardson A, Little P, Yardley L (2018). Understanding acceptability of and engagement with Web-based interventions aiming to improve quality of life in cancer survivors: A synthesis of current research. Psychooncology.

[34] Ruland CM, Andersen T, Jeneson A, Moore S, Grimsbø Gro H, Børøsund Elin, Ellison MC (2013). Effects of an internet support system to assist cancer patients in reducing symptom distress: a randomized controlled trial. Cancer Nurs.

[35] Michie S, Yardley L, West R, Patrick K, Greaves F (2017). Developing and Evaluating Digital Interventions to Promote Behavior Change in Health and Health Care: Recommendations Resulting From an International Workshop. J Med Internet Res.

[36] Coolbrandt A, Wildiers H, Aertgeerts B, Dierckx de Casterlé Bernadette, van Achterberg T, Milisen K (2018). Systematic development of CHEMO-SUPPORT, a nursing intervention to support adult patients with cancer in dealing with chemotherapy-related symptoms at home. BMC Nurs.

[37] Paige SR, Stellefson M, Krieger JL, Anderson-Lewis C, Cheong J, Stopka C (2018). Proposing a Transactional Model of eHealth Literacy: Concept Analysis. J Med Internet Res.

[38] Gasson S (2003). Human-Centered vs User-Centered Approaches to Information System Design. Journal of Information Technology Theory and Application.

[39] Matheson GO, Pacione C, Shultz RK, Klügl Martin (2015). Leveraging human-centered design in chronic disease prevention. Am J Prev Med.

[40] Ivankova NV, Creswell Jw, Stick Sl (2016). Using Mixed-Methods Sequential Explanatory Design: From Theory to Practice. Field Methods.

[41] Visser FS, Stappers PJ, van der Lugt R, Sanders EB (2005). Contextmapping: experiences from practice. CoDesign.

[42] Eyles H, Jull A, Dobson R, Firestone R, Whittaker R, Te Morenga L, Goodwin D, Mhurchu CN (2016). Co-design of mHealth Delivered Interventions: A Systematic Review to Assess Key Methods and Processes. Curr Nutr Rep.

[43] Sandelowski M (2000). Whatever happened to qualitative description?. Res Nurs Health.

[44] Chafe R (2017). The Value of Qualitative Description in Health Services and Policy Research. Healthc Policy.

[45] Haase K, Avery J, Bryant-Lukosius D, Kryzanowska Monika, Kukretti Vishal, Liu Geoffrey, Mayo Samantha J, Jones Jennifer, Howell Doris (2021). Patient and clinician perspectives of desired features for a web-based self-management program (icanmanage.ca): exposing patients "hard work" of managing acute cancer. Support Care Cancer.

[46] Nielsen L, Soegaard M, Rikke FD (2018). Personas. The Encyclopedia of Human-Computer Interaction (2nd edn).

[47] Symptom Management Guidelines. British Columbia Cancer Agency.

[48] Managing symptoms, side-effects and well-being. Health care professional version. Ontario Health.

[49] Managing symptoms, side-effects and well-being. Health care professional version (patient guides). Ontario Health.

[50] Bandura A (1982). Self-efficacy mechanism in human agency. American Psychologist.

[51] Bandura A (1977). Self-efficacy: Toward a unifying theory of behavioral change. Psychological Review.

[52] Articulate Storyline 360. Articulate.

[53] Moodle Learning Platform. Moodle Docs.

[54] ASCO Doctor-approved information. Cancer.Net.

[55] Howell D (2018). Supported self-management for cancer survivors to address long-term biopsychosocial consequences of cancer and treatment to optimize living well. Curr Opin Support Palliat Care.

[56] Lewis JR (2006). Sample sizes for usability tests. interactions.

[57] Wharton C, Riemann J, Lewis C, Polson P, Neilson J (1994). The Cognitive Walkthrough Method: A Practitioners Guide. Usability Inspection Methods.

[58] Lewis Jr (2018). The System Usability Scale: Past, Present, and Future. International Journal of Human–Computer Interaction.

[59] Bashi N, Fatehi F, Mosadeghi-Nik M, Askari MS, Karunanithi M (2020). Digital health interventions for chronic diseases: a scoping review of evaluation frameworks. BMJ Health Care Inform.

[60] Yardley L, Choudhury T, Patrick K, Michie S (2016). Current Issues and Future Directions for Research Into Digital Behavior Change Interventions. Am J Prev Med.

[61] Gambalunga F, Iacorossi L, Notarnicola I, Serra V, Piredda M, De Marinis MG (2020). Mobile Health in Adherence to Oral Anticancer Drugs: A Scoping Review. Comput Inform Nurs.

[62] Fridriksdottir N, Gunnarsdottir S, Zoëga S, Ingadottir B, Hafsteinsdottir EJG (2018). Effects of web-based interventions on cancer patients' symptoms: review of randomized trials. Support Care Cancer.

[63] Kanera IM, Bolman CAW, Willems RA, Mesters I, Lechner L (2016). Lifestyle-related effects of the web-based Kanker Nazorg Wijzer (Cancer Aftercare Guide) intervention for cancer survivors: a randomized controlled trial. J Cancer Surviv.

[64] Institute of Medicine (2013). Delivering High-Quality Cancer Care: Charting a New Course for a System in Crisis.

[65] Howell D, Powis M, Kirkby R, Amernic H, Moody L, Bryant-Lukosius D, O'Brien MA, Rask S, Krzyzanowska MK (2022). Improving the quality of self-management support in ambulatory cancer care: a mixed-method study of organisational and clinician readiness, barriers and enablers for tailoring of implementation strategies to multisites. BMJ Qual Saf.

[66] Levesque JV, Lambert SD, Girgis A, Turner J, McElduff P, Kayser K (2015). Do men with prostate cancer and their partners receive the information they need for optimal illness self-management in the immediate post-diagnostic phase?. Asia Pac J Oncol Nurs.

[67] Viola A, Panigrahi G, Devine KA (2020). Digital interventions for adolescent and young adult cancer survivors. Curr Opin Support Palliat Care.

[68] Haase KR, Sattar S, Hall S, McLean B, Wills A, Gray M, Kenis C, Donison V, Howell D, Puts M (2021). Systematic review of self-management interventions for older adults with cancer. Psychooncology.

[69] Lövdén Martin, Fratiglioni L, Glymour M, Lindenberger U, Tucker-Drob E (2020). Education and Cognitive Functioning Across the Life Span. Psychol Sci Public Interest.

[70] Georgeff M (2014). Digital technologies and chronic disease management. Aust Fam Physician.

[71] Slev VN, Mistiaen P, Pasman HRW, Verdonck-de Leeuw Irma M, van Uden-Kraan Cornelia F, Francke AL (2016). Effects of eHealth for patients and informal caregivers confronted with cancer: A meta-review. Int J Med Inform.

[72] Adriaans Danielle Jm, Dierick-van Daele Angelique Tm, van Bakel Marc Johannes Hubertus Maria, Nieuwenhuijzen Grard Ap, Teijink Joep Aw, Heesakkers Fanny Fbm, van Laarhoven Hanneke Wm (2021). Digital Self-Management Support Tools in the Care Plan of Patients With Cancer: Review of Randomized Controlled Trials. J Med Internet Res.

