# Supplementary material for: A Web-Based Cancer Self-Management Program (I-Can Manage) Targeting Treatment Toxicities and Health Behaviors: Human-Centered Co-design Approach and Cognitive Think-Aloud Usability Testing
Source: JMIR Cancer. 2023 Jul 21;9:e44914. doi: 10.2196/44914 (PMC10403801; doi:10.2196/44914)
Supplement: Multimedia Appendix 1 [file cancer_v9i1e44914_app1.docx]

**Supplement 1: Summarized Themes of Experience, Defining Moments and Needs from Persona and Cancer Journey Mapping**

| Phases | Diagnosis | Decision-making | Treatment | Post-Treatment |
| --- | --- | --- | --- | --- |
| Moments | Hearing the diagnosis was life altering; a new reality.  Processing the news and adjusting emotionally. Worried about telling family and their reaction.  Details about my cancer.  Prognosis information and fears about dying.  I wasn’t expecting this! | How to research cancer and internet searching  Decisions about how much to share with others.  Making decisions and next steps.  Understanding prognosis information.  Expected outcomes of treatment and options. | Mandatory chemo class-daunting too many patients, some were sick, Why do I have to be here?  Face to face teaching for radiotherapy.  Unable to anticipate impact of side-effects on functioning i.e. peripheral neuropathy | Am I cancer free? It’s not really over but told “you are done”.  Ongoing disease management.  Managing new conditions; fear of recurrence.  Managing psychological issues like depression, post-traumatic stress and pain management  Adjustment to new normal. |
| Caregivers | What does it mean to be a caregiver, how much involvement?  Need discussion around caregivers.  How can family support me?  Support for family to adjust.  Caregiver burnout and anxiety and depression. | Who can support me if family not available?  Who can help me to figure out how to talk to my kids?; I don’t know how to tell them; should I tell them?; How can I get help with this?  What resources can I access (social worker, nurse, psychosocial team). | What can family members do to help manage effects of treatment? | Caregivers need to understand slow recovery of health |
| Know/Learn/Do | Need to know what to expect; further tests; final diagnosis.  Where are the cancer support groups?  How to avoid unsolicited info/advice and assumptions.  Differences between home health systems and regular health system?  What’s next? I wish I had a crystal ball; how does this play out?  You have to be organized- taking own notes on ipad during appointment.  Maintaining hope. | Access to reliable, credible trustworthy information.  How do I weigh out benefits/side-effects of treatments?  How to research the options; informed consent  Access to other cancer patients’ stories.  Health literacy.  Care Requirements  Understanding where my prognosis /stats fits in with my care.  What questions should I ask my oncology team  Weighing options, how do I decide what is right for me? | Experience of Chemo, learn process, look for pattern/timing to treatment, changes to body, adjust to side effects of drugs  Treatment side effects, what is normal, what to expect, how to manage.  Knowing how to persevere; advocate for quality care.  Who to talk to and when?  Process all of the  information, how do I make sense of it?  What are your expectations? Next steps?  How long do I have?  Patient education 101; a lot!  Tracking actions, symptoms, daily timing;  Who do I call when I have side effects especially after hours?  What health care resources? | What are my community supports; services available i.e. hospice/palliative care.  Holding on to quality of life/live well with cancer.  Learn how to adjust to new body & emotional state.  Learning about on-going monitoring efforts  Tests? Long term? What’s next?  Side effects, long term care? |
| Challenges | Complexity of information, overwhelmed, poor communication for clinicians. Cancer will not define me.  Knowing what you don’t know; huge learning curve/accelerated rate to catch up with diagnosis. Conflicting experience of others; each patients experience is unique.  Access to information (practical and medical); information overload need for filtering; stumbling no clear direction; lack of control when searching for information online; credibility of information sources; ripple effects of treatment and not knowing about them; speed/rate that things happen  Continuity in providers, lacking family physician to pull reports, logistics together | Indecision and pressure to make decisions.  Multiple clinicians; need to keep records.  Finances; cost of drugs; getting to treatments; health benefits; economic-job loss; day care costs.  Logistical support even just getting to treatment; practicalities.  Access to psychosocial support; variable access to psychosocial support/hospice/palliative care.  Power of attorney, estate planning, wills.  Constantly changing treatment recommendations; questions to ask.  Understanding where I fit in. My past culture blended with new culture and my lack of understanding health literacy from that intersection. | Figuring out what behaviour as patient is acceptable (being own advocate).  How much can my body take? (knowing thresholds -when to stop), not knowing who to contact re issues.  Expectations of self (not having same stamina) expectation of others (family not realizing you need support).  Knowing which provider to go to (navigating)  Can I work?  Social support  Who looks after kids?  How to manage side effects?  TIME, all of a sudden all of my time is sucked up with “surviving this” ; Where does family, job fit in? Long waits, wasted time waiting for appointments, treatments. | Moving from survivorship to new normal  Going to GP without any concrete plan of care  Ongoing physical limitations  Coordination of care between providers (oncologist and GP)  Understanding why I am feeling this way  Getting complete information of all issues  Getting reduced access to services especially mental health (getting dropped from Cancer Centre)  What happens if it comes back?  Will it come back?  Safety net of relationship with professionals.  After care, possible symptoms. |
| Thoughts and Feelings | Why me, confusing, overwhelmed, anxiety, depression, lack of trust, fear and stress; seeking spiritual support;  Most chaotic emotional stage, pre-diagnosis; what is okay to feel/do/act; don’t patronize me; don’t want to be pitied; anger why me and why not me  Uncertainty, scared.  Still want my wife, kids to feel loved, cared for – not worried etc.  Anticipating angst, is this going to kill me? What has my new normal become? | What can I do to help myself-eating, exercise, complementary; stress, quality of care, fear and anxiety of secondary infections; appearance; hair loss; weight gain/loss; loss of self-esteem and self-confidence.  What should I do?  Scared, confused. What does this mean for me? How to process all the information and to make sense of my unique situation?  What is going to happen to my long-term plans to provide for my kid’s future; Who will provide for them?  No influence on kids -What can my legacy be if I am not here? | Persevering and endurance  Fear of unknown- linked to ripple effects of treatment  What’s important?  How to care for comorbidities?  Don’t want to waste my time on small unpleasant things, more sensitive to not wasting time, doing things you want to do.  Caregiver support limited  Perseverance and endurance; fear of treatment throughout. | Anxiety, depression. Burnout  Empowerment to try new things.  Crash and burn  Fear of recurrence  Post-traumatic stress symptoms  Isolation after treatment (care team, family and friends)  Pedestal syndrome, being deemed a survivor, thriver, etc  Are you cured?  Your perspective /others perspective  What’s next? |
