# Supplementary figures and images for: A Web-Based Cancer Self-Management Program (I-Can Manage) Targeting Treatment Toxicities and Health Behaviors: Human-Centered Co-design Approach and Cognitive Think-Aloud Usability Testing
Source: JMIR Cancer. 2023 Jul 21;9:e44914. doi: 10.2196/44914 (PMC10403801; doi:10.2196/44914)

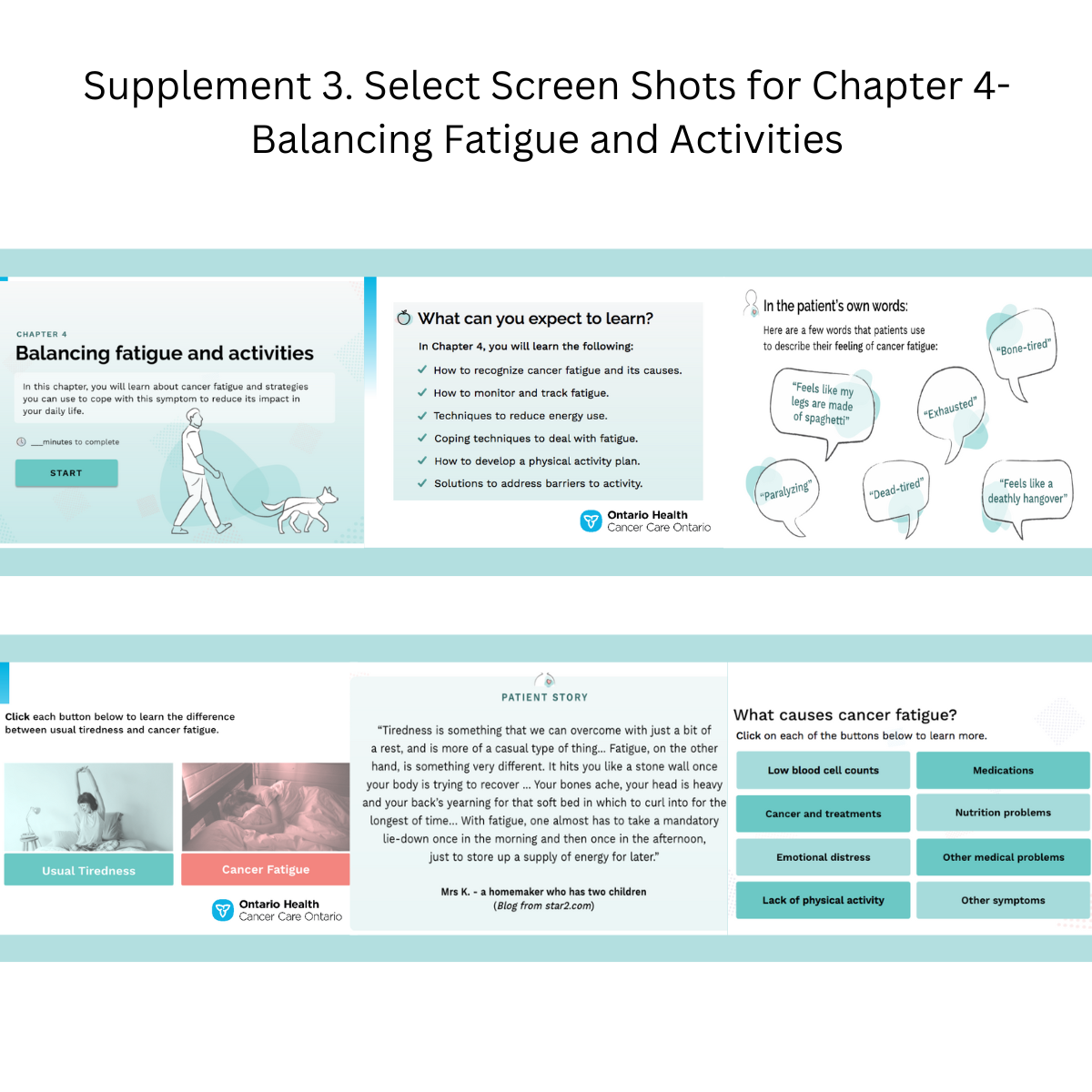

Supplement: Multimedia Appendix 2 [file cancer_v9i1e44914_app2.png]

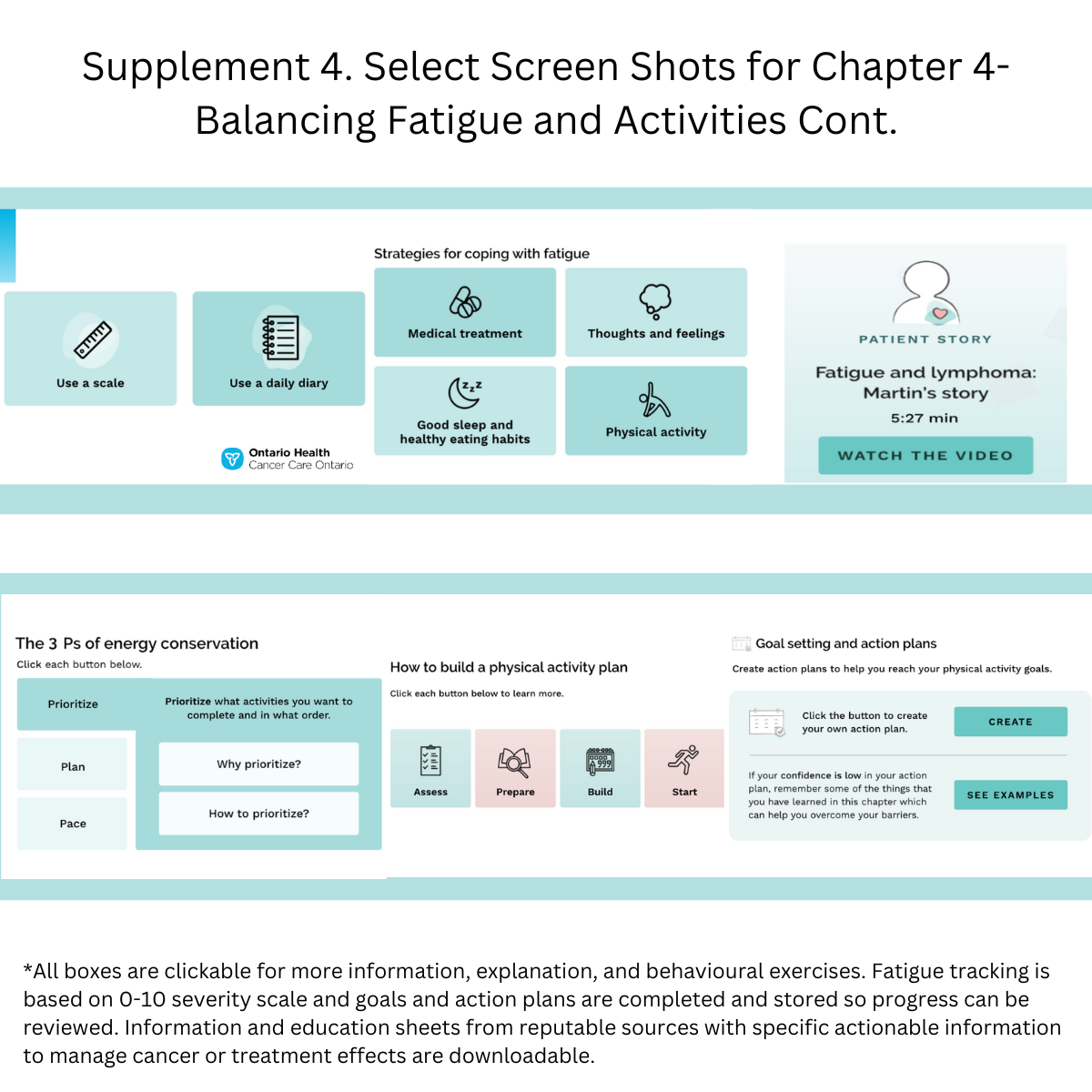

Supplement: Multimedia Appendix 3 [file cancer_v9i1e44914_app3.png]

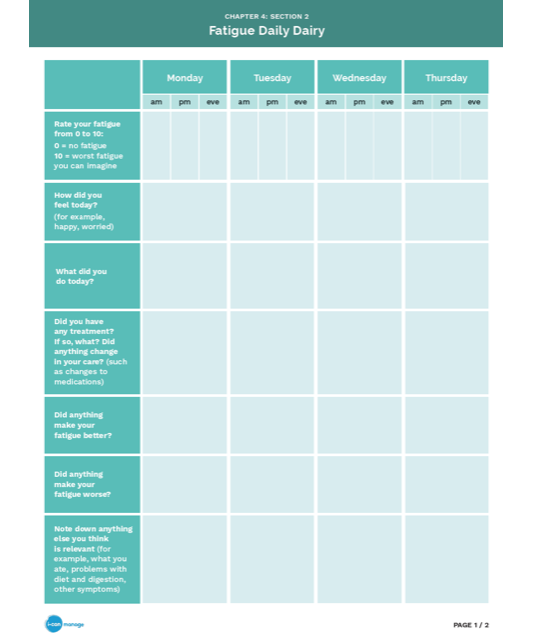

Supplement: Multimedia Appendix 4 [file cancer_v9i1e44914_app4.png]

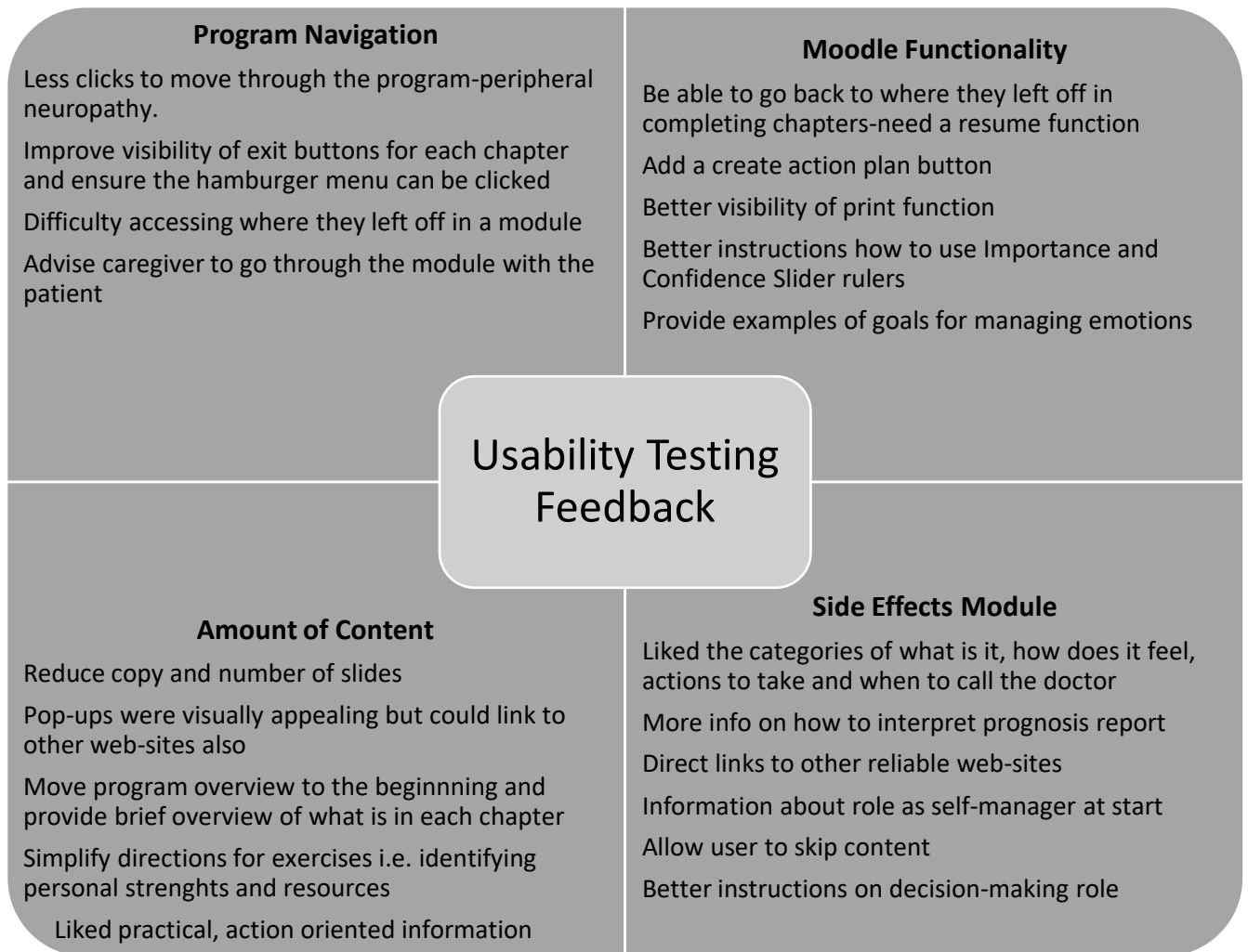

Supplement 2: Summary of Refinement for Usability Feedback to Inform Program

Supplement: Multimedia Appendix 6 [file cancer_v9i1e44914_app6.pdf]
