# Supplementary material for: A Web-Based Cancer Self-Management Program (I-Can Manage) Targeting Treatment Toxicities and Health Behaviors: Human-Centered Co-design Approach and Cognitive Think-Aloud Usability Testing
Source: JMIR Cancer. 2023 Jul 21;9:e44914. doi: 10.2196/44914 (PMC10403801; doi:10.2196/44914)
Supplement: Multimedia Appendix 5 [file cancer_v9i1e44914_app5.pdf]

Sleep Hygiene Strategies

Create a Comfortable Sleep Environment

1. Create a comfortable sleep environment.
   For example, a supportive mattress and fresh, comfortable bedding.
2. Do not have your bedroom too hot or cold, minimize noise, and block out light.

Relax

1. Try doing something to relax your body and mind before going to bed.
2. Try taking a hot bath **90 minutes** before you plan to go to bed or try a relaxation exercise (see Calm Breathing and Progressive Muscle Relaxation), meditation or listening to calming music.

Snack

Although a heavy meal late in the evening can disrupt sleep, a healthy, light snack in the evening can improve sleep.

1. Try eating light cheese and crackers, turkey or bananas, or drink a warm glass of milk.
2. Avoid heavy, spicy or sugary foods.

Don't Smoke Before Bed

1. Try to avoid smoking at least **four hours** before bedtime as it can interfere with a good night's sleep.

Keep Moving

People who exercise tend to have more restful sleep. Exercising for at least **30 minutes, three times a week,** can improve your sleep.

1. Exercise in the late afternoon or early evening is the best.
2. Avoid exercise if it is less than **two hours** before bed.

Fixed Bedtime Routine

A bedtime routine tells your body that it is time to sleep.

1. Establish a set routine that you follow every night.
   For example, have a hot bath, put on your pajamas, brush your teeth, listen to soft music and read on the couch until you start to feel sleepy.
2. Only go to bed when you are feeling sleepy.

Fixed Waking Time

1. Try waking up at the same time every day (even on weekends) no matter how well or how poorly you slept. This way your body will begin to get used to a regular sleep rhythm.
2. Don’t force yourself into bed at a particular time if you are not feeling sleepy. You will only lie awake in bed, frustrated that you cannot sleep.

Bedroom

1. Your bed should be used strictly for sleeping (sex is the only exception).
2. Try to avoid reading, watching television, working, or studying in bed. These activities keep your mind active, which gets in the way of sleeping.

Avoid Alcohol

Although you may think that alcohol will help you fall asleep, it interferes with sleep later in the evening.

1. Try to avoid consuming alcohol at least **four hours** before bed.

Avoid Caffeine

1. Avoid drinking caffeine at least **four hours** before bedtime. This includes coffee, some teas, soft drinks, and chocolate. Caffeine is a stimulant and it can keep you awake.

Natural Light

1. Try to spend some time outdoors or in natural light every day. Getting some sunlight early in the day can be helpful for setting your body's natural wake and sleep cycle.

Shorten your Rest or Naps

Naps can interfere with normal sleep cycles.

1. If you are having trouble sleeping, avoid taking naps. That way, your body will be more tired when it is bedtime.
2. Try taking short rest and naps during the day, which may be essential to manage fatigue.

Get out of Bed

1. **If you cannot fall asleep after 20 to 30 minutes, get out of bed and do something boring.** For example, read the manual on how to program your clock radio, read the sports section of the newspaper if you are not a sports fan.
2. **Try relaxing.** For example, meditate, listen to calm music, or have a warm decaffeinated drink.

Don't Worry

Leave your worries about things like work, school, health and relationships out of the bedroom.

1. **Try scheduling a "worry time" earlier in the evening to deal with your worries.**
2. **If you wake up in the middle of the night worrying, try writing down your worries and tell yourself that you will address them in the morning.**

TIP: **Worrying about not sleeping does not help.** This just makes it more likely that you will not sleep.

- Let go of your belief that you have to get eight hours of sleep or you cannot function.
- Stop looking at the clock and stop trying to make yourself fall sleep. It will happen when it happens.
